# Secondhand smoke increases the risk of developing kidney stone disease

**DOI:** 10.1038/s41598-021-97254-y

**Published:** 2021-09-06

**Authors:** Chien-Heng Chen, Jia-In Lee, Jhen-Hao Jhan, Yung-Chin Lee, Jiun-Hung Geng, Szu-Chia Chen, Chih-Hsing Hung, Chao-Hung Kuo

**Affiliations:** 1 Department of Pathology, Kaohsiung Municipal Siaogang Hospital, Kaohsiung, Taiwan; 2 Department of Psychiatry, Kaohsiung Medical University Hospital, Kaohsiung Medical University, Kaohsiung, Taiwan; 3 Department of Urology, Kaohsiung Municipal Siaogang Hospital, Kaohsiung, Taiwan; 4 Department of Urology, Kaohsiung Medical University Hospital, Kaohsiung, Taiwan; 5 Kaohsiung Medical University, Kaohsiung, Taiwan; 6 Department of Internal Medicine, Kaohsiung Municipal Siaogang Hospital, Kaohsiung Medical University, Kaohsiung, Taiwan; 7 Division of Nephrology, Department of Internal Medicine, Kaohsiung Medical University Hospital, Kaohsiung Medical University, Kaohsiung, Taiwan; 8 Faculty of Medicine, College of Medicine, Kaohsiung Medical University, Kaohsiung, Taiwan; 9 Research Center for Environmental Medicine, Kaohsiung Medical University, Kaohsiung, Taiwan; 10 Department of Pediatrics, Kaohsiung Medical University Hospital, Kaohsiung Medical University, Kaohsiung, Taiwan; 11 Department of Pediatrics, Kaohsiung Municipal Siaogang Hospital, Kaohsiung Medical University, Kaohsiung, Taiwan; 12 Division of Gastroenterology, Department of Internal Medicine, Kaohsiung Medical University Hospital, Kaohsiung Medical University, Kaohsiung, Taiwan

**Keywords:** Kidney diseases, Risk factors, Health care, Disease prevention, Health policy, Public health

## Abstract

Research indicates smoking increases the risk of various kidney diseases, although the risk of developing kidney stone disease in non-smokers exposed to secondhand smoke is unknown. This study analyzed a total of 19,430 never-smokers with no history of kidney stone disease who participated in the Taiwan Biobank from 2008 to 2019. They were divided into two groups by secondhand smoke exposure; no exposure and exposure groups; the mean age of participants was 51 years, and 81% were women. Incident kidney stone development was observed in 352 (2.0%) and 50 (3.3%) participants in the no exposure and exposure groups during a mean follow-up of 47 months. The odds ratio (OR) of incident kidney stone was significantly higher in the exposure group than the no exposure group [OR, 1.64; 95% confidence interval (95% CI) 1.21 to 2.23]. Participants with > 1.2 h per week exposure were associated with almost twofold risk of developing kidney stones compared with no exposure (OR, 1.92; 95% CI 1.29 to 2.86). Our study suggests that secondhand smoke is a risk factor for development of kidney stones and supports the need for a prospective evaluation of this finding.

## Introduction

Kidney stone disease (KSD) is a global health problem. The lifetime risk of KSD in Taiwan is around 10%^1,2^, which is similar to North America and Europe^3^. KSD is related to an increased risk of urinary tract infection and chronic kidney disease^4^, and may also result in life-threatening diseases, including septicaemia. Most stones form by a combination of genetics and environmental factors, so identifying modifiable factors and applying primary prevention is urgently needed to reduce the burden of KSD.

Smoking injures bodily health and causes damage to organs, including the kidneys^5^. Reports have shown that smoking is related to a higher risk of chronic kidney disease^6^, renal cancer^7,8^ and kidney stones^9^; however, little is known about the relationship between secondhand smoke (SHS) and kidney stone development. In addition, SHS could be comprised of even more toxic chemicals than those found in tobacco smoke, postulating that SHS could be harmful^10,11^. It is important to understand the impact of SHS on kidney health, which could help enforce restrictions of smoking in our living environment. Based on previously limited results and the lack of a large cohort study follow-up on this issue, the aim of the present study was to investigate the association between SHS exposure and the risk of KSD in a longitudinal data of 19,430 participants from the Taiwan Biobank (TWB) study.

## Results

### Baseline characteristics

The baseline profiles of the 19,430 included participants are summarized in Table 1. The mean age was 51 ± 10 years; the majority of subjects (81%) were female; and a total of 17,905 (92%) subjects were included in the no exposure group. Among 1525 participants exposed to SHS, the median exposure hours per week were 1.2 (interquartile range 0.5 to 5). Participants were more exposed to SHS at home (n = 974, 65%) than at work (n = 598, 39%), with SHS exposure groups tending to have higher prevalence of alcohol consumption, lower prevalence of physical activity, were less married, and had higher body mass index (BMI) than those in the no exposure group.

**Table 1:** Clinical characteristics of the study participants classified by exposure of secondhand smoke.

Characteristics	Total, n = 19,430	Secondhand smoke exposure		*p* value
		No exposure, n = 17,905	Exposure, n = 1525	
**Demographic data**				
Age, yr	51 ± 10	51 ± 10	48 ± 10	0.387
Women	15,644 (81)	14,482 (81)	1162 (76)	< 0.001
BMI, kg/m^2^	23.7 ± 3.5	23.7 ± 3.5	24.2 ± 3.8	0.001
Alcohol status, ever	571 (3)	458 (3)	113 (7)	< 0.001
Physical activity, yes	8987 (46)	8422 (47)	565 (37)	< 0.001
Married, yes	17,693 (91)	16,337 (91)	1356 (89)	0.002
Systolic BP, mm Hg	117 ± 18	116 ± 18	116 ± 18	0.363
Diastolic BP, mm Hg	72 ± 11	72 ± 11	72 ± 11	0.118
**Comorbidities**				
Hypertension	2174 (11)	2012 (11)	162 (11)	0.465
Diabetes	890 (5)	818 (5)	72 (5)	0.784
Dyslipidemia	1260 (7)	1170 (7)	90 (6)	0.335
**Laboratory data**				
eGFR, ml/min per 1.73 m^2^	105 ± 24	105 ± 24	106 ± 25	0.085
Hemoglobin, g/dl	13.4 ± 1.5	13.4 ± 1.4	13.4 ± 1.5	0.003
Albumin, g/dl	4.5 ± 0.2	4.5 ± 0.2	4.6 ± 0.2	0.004
Fasting glucose, mg/dl	95 ± 19	95 ± 19	96 ± 22	< 0.001
Hemoglobin A1c, %	5.7 ± 0.7	5.7 ± 0.7	5.7 ± 0.9	0.001
Total cholesterol, mg/dl	196 ± 36	196 ± 36	194 ± 36	0.489
Triglyceride, mg/dl	107 ± 75	106 ± 73	110 ± 92	0.001
HDL cholesterol, mg/dl	56 ± 13	56 ± 13	55 ± 13	0.152
LDL cholesterol, mg/dl	121 ± 32	122 ± 32	121 ± 31	0.881
Uric acid, mg/dl	5.2 ± 1.3	5.2 ± 1.3	5.4 ± 1.4	< 0.001

*BMI* body mass index, *BP* blood pressure, *eGFR* estimated glomerular filtration rate, *HDL* high-density lipoproteins, *LDL* low-density lipoproteins.

### Association between SHS and the development of KSD

A total of 402 participants (2.1% of the study population) had development of KSD during a mean follow-up duration of 47 ± 14 months. In univariate analysis, age, gender, married status, BMI, systolic blood pressure (SBP), diastolic blood pressure (DBP), history of hypertension, history of dyslipidemia, serum hemoglobin, albumin, fasting glucose, hemoglobin A1c, triglyceride, high-density lipoprotein cholesterol and uric acid were significantly associated with the development of KSD (Table 2). The risk for incident kidney stone development was also significantly higher in the SHS exposure group than in the no exposure group [odds ratio (OR), 1.69; 95% confidence interval (CI) 1.25 to 2.28, *p* value = 0.001]. After adjusting for confounders, male gender, higher DBP, history of hypertension and SHS exposure were four independent risk factors for the development of KSD (Table 3). Comparing the SHS exposure group with the no exposure group, the OR for developing KSD showed significant increase in the exposure group (OR, 1.64; 95% CI 1.21 to 2.23, *p* value = 0.002). The association remained significant in models with additional adjustment for daily water intake in a subgroup of 8850 participants with adequate information (OR, 2.05; 95% CI 1.40 to 2.99, *p* value < 0.001) (Supplementary Table S1).

**Table 2:** Parameters associated with incident kidney stone in univariable binary logistic analysis in all study participants (n = 19,430).

Parameters	Odds ratio (95% CI)	*p*
Age (per 1 year)	1.011 (1.001 to 1.021)	0.032
Women (vs. men)	0.415 (0.337 to 0.510)	< 0.001
Body mass index (per 1 kg/m^2^)	1.065 (1.038 to 1.092)	< 0.001
Alcohol status, ever (vs. never)	1.472 (0.899 to 2.411)	0.124
Physical activity, yes (vs. no)	1.011 (0.829 to 1.232)	0.915
Married, yes (vs. no)	1.631 (1.068 to 2.492)	0.024
Systolic blood pressure (per 1 mmHg)	1.013 (1.008 to 1.018)	< 0.001
Diastolic blood pressure (per 1 mmHg)	1.031 (1.022 to 1.040)	< 0.001
Hypertension, yes (vs. no)	1.913 (1.486 to 2.462)	< 0.001
Diabetes mellitus, yes (vs. no)	1.271 (0.830 to 1.947)	0.270
Dyslipidemia, yes (vs. no)	1.753 (1.271 to 2.418)	0.001
eGFR (per 1 ml/min/1.73 m^2^)	0.996 (0.992 to 1.001)	0.085
Hemoglobin (per 1 g/dl)	1.307 (1.216 to 1.403)	< 0.001
Albumin (per 1 g/dl)	1.851 (1.197 to 2.862)	0.006
Fasting glucose (per 1 g/dl)	1.005 (1.001 to 1.009)	0.009
Hemoglobin A1c (per 1%)	1.141 (1.028 to 1.267)	0.013
Total cholesterol (per 1 mg/dl)	1.001 (0.998 to 1.003)	0.704
Triglyceride (per 1 mg/dl)	1.002 (1.001 to 1.002)	< 0.001
HDL cholesterol (per 1 mg/dl)	0.983 (0.975 to 0.991)	< 0.001
LDL cholesterol (per 1 mg/dl)	1.003 (0.999 to 1.006)	0.109
Uric acid (per 1 mg/dl)	1.228 (1.146 to 1.315)	< 0.001
Secondhand smoke exposure, yes (vs. no)	1.690 (1.251 to 2.284)	0.001

*BP* blood pressure, *eGFR* estimated glomerular filtration rate, *HDL* high-density lipoproteins, *LDL* low-density lipoproteins, *CI* confidence interval.

**Table 3:** Parameters associated with incident kidney stone in multivariate binary logistic analysis in all study participants (n = 19,430).

Parameters	Odds ratio (95% CI)	*p*
Age (per 1 year)	1.005 (0.993 to 1.017)	0.385
Women (vs. men)	0.585 (0.438 to 0.782)	< 0.001
Body mass index (per 1 kg/m^2^)	1.023 (0.991 to 1.057)	0.161
Alcohol status, ever (vs. never)	–	–
Physical activity, yes (vs. no)	–	–
Married, yes (vs. no)	1.530 (0.983 to 2.381)	0.060
Systolic blood pressure (per 1 mmHg)	0.995 (0.986 to 1.004)	0.297
Diastolic blood pressure (per 1 mmHg)	1.021 (1.007 to 1.035)	0.004
Hypertension, yes (vs. no)	1.383 (1.037 to 1.845)	0.028
Diabetes mellitus, yes (vs. no)	–	–
Dyslipidemia, yes (vs. no)	1.377 (0.979 to 1.937)	0.066
eGFR (per 1 ml/min/1.73 m^2^)	–	–
Hemoglobin (per 1 g/dl)	1.086 (0.991 to 1.190)	0.079
Albumin (per 1 g/dl)	1.178 (0.736 to 1.885)	0.495
Fasting glucose (per 1 g/dl)	0.999 (0.991 to 1.007)	0.824
Hemoglobin A1c (per 1%)	1.027 (0.831 to 1.269)	0.806
Total cholesterol (per 1 mg/dl)	–	–
Triglyceride (per 1 mg/dl)	1.000 (0.999 to 1.002)	0.432
HDL cholesterol (per 1 mg/dl)	0.998 (0.989 to 1.007)	0.686
LDL cholesterol (per 1 mg/dl)	–	–
Uric acid (per 1 mg/dl)	0.998 (0.914 to 1.090)	0.969
Secondhand Smoke Exposure, yes (vs. no)	1.643 (1.209 to 2.234)	0.002

Multivariable model: adjustment for age, sex, marital status, body mass index, systolic blood pressure, diastolic blood pressure, history of hypertension, history of dyslipidemia, hemoglobin, Hemoglobin A1c, serum fasting glucose, triglyceride, high-density lipoprotein cholesterol, serum albumin, and serum uric acid.

*BP* blood pressure, *eGFR* estimated glomerular filtration rate, *HDL* high-density lipoproteins, *LDL* low-density lipoproteins, *CI* confidence interval.

### Association between SHS frequency and the development of KSD

To further evaluate the association between incident KSD and frequency of SHS exposure, we divided participants into three groups: no exposure, ≦ 1.2 h per week, and > 1.2 h per week according to the median exposure time. After adjusting for confounders, those exposed to SHS ≦ 1.2 h per week (OR, 1.40; 95% CI 0.90 to 2.18, *p* value = 0.138) or > 1.2 h per week (OR, 1.92; 95% CI 1.29 to 2.86, *p* value = 0.002) were associated with a higher risk for incident KSD compared with those not exposed to SHS (Table 4). Similar results were found when additional adjusting daily water intake (N = 8850) as shown in Supplementary Table S2.

**Table 4:** Relative risk for incident kidney stone disease according to frequency of secondhand smoke.

Secondhand smoke frequency	No. of cases (%)	Number at risk	Adjusted odds ratio (95% CI)	*p* value
No exposure	352 (2.0)	17,905	1.00 (Reference)	–
≦ 1.2 h per week	22 (2.8)	786	1.40 (0.90 to 2.18)	0.138
> 1.2 h per /week	28 (3.8)	739	1.92 (1.29 to 2.86)	0.002

Multivariable model: adjustment for age, sex, marital status , body mass index, systolic blood pressure, diastolic blood pressure, history of hypertension, history of dyslipidemia, hemoglobin, Hemoglobin A1c, serum fasting glucose, triglyceride, high-density lipoprotein cholesterol, serum albumin, and serum uric acid.

*CI* confidence interval.

## Discussion

In this longitudinal study of a representative sample of the Taiwan population, SHS was significantly associated with a higher risk of incident KSD after adjustment of covariates. In addition, participants with > 1.2 h per week exposure were associated with almost twofold risk of developing kidney stones compared with no exposure.

According to the World Health Organization (WHO), 40% of children, 35% of female non-smokers, and 33% of male non-smokers were exposed to SHS worldwide in 2004^12^. Moreover, convincing evidence claims that exposure to SHS causes more than 880,000 deaths worldwide each year^12^. These findings have induced policy makers to take action on protecting the population against exposure to SHS. In Taiwan, the proportion of participants who were exposed to SHS was 26.5% in 2008^13^, and as a result of the amendment to the Tobacco Hazards Prevention Act in 2009, the proportion decreased substantially^14^, being 8% in our cohort. Although the number of individuals exposed to SHS decreased between 1990 and 2016^15^, there is still an urgent need to understanding the harmful effects associated with SHS. Our research builds upon a large-scale community-based cohort in Taiwan with information about lifestyle, medical history and exposed environments, which provide an ideal platform for research to explore the causes of common diseases, including KSD. To our knowledge, this is the first study to demonstrate a possible influence of SHS on the development of KSD.

Previous, evidence reveals that exposure to SHS can cause a variety of health issues in non-smokers, such as cardiovascular diseases^16^, respiratory diseases^16^, lung cancer^16^, and kidney diseases^17^. A large screened population of more than 130,000 healthy Asian men showed a history of SHS exposure was associated with a higher risk for chronic kidney disease^17^. In addition, Omoloja et al. found that SHS exposure was an independent risk factor for nephrotic range proteinuria in children (OR, 2.64; 95% CI 1.08 to 6.42)^18^. These findings indicate that exposure to SHS might cause glomerular dysfunction and a decreased output of urine, both of which might induce urolithiasis^19–21^.

Consistent with the hypothesis that SHS exposure could injure the kidneys, an association between SHS and KSD was demonstrated, with the finding pointing to the fact that SHS could be as dangerous as smoking in developing kidney stones^9,22–24^. Liu et al. analyzed 354 cases and 354 age- and sex-matched healthy controls and found current cigarette smoking was an independent risk factor for the development of kidney stones (OR 1.66; 95% CI 1.11 to 2.50)^22^. Similar results were seen among 102 cases in Iran (OR 2.06; 95% CI 1.06 to 4.01)^9^, and 181 cases in Japan (OR 4.29; 95% CI 2.68 to 6.86)^23^. In line with these studies, this study found that non-smokers exposed to SHS had a significant increase of nephrolithiasis risk in a multivariate binary logistic analysis (OR 1.64; 95% CI 1.21 to 2.23). Although, there is no direct comparison of the risk of developing kidney stones between smokers and non-smokers with SHS exposure, the toxic chemicals inhaled by first-hand smokers and secondhand smokers are similar, and the adverse health effects are similar as well^25^.

In the present study, a subgroup analysis stratified by frequency of SHS exposure found non-smokers exposed to SHS ≦ 1.2 h per week had a 1.4-fold higher risk of developing kidney stones than those not exposed; moreover, participants who were exposed for more than 1.2 h per week were associated with an almost twofold risk of developing kidney stones compared with no exposure. The current literature has shown there is no risk-free level of SHS exposure^16,26^. Studies also revealed that even brief (minutes to hours) exposure can be as harmful to health as chronic active smoking^27,28^. Our findings suggest that even infrequent exposure to SHS could lead to a higher rate of stone formation, which may be helpful for public health and clinical care. If SHS is related to subsequent kidney stone diseases, a comprehensive smoke-free policy could be adopted to prevent the development of stones to protect non-smokers, and this is the only way to fully protect the health of this group^16^.

Although the exact mechanism of SHS exposure-associated renal stone formation is undetermined, it is speculated that exposure to SHS might produce similar renal damage to that seen among active smokers. Based on previous research, non-smokers exposed to SHS will inhale 80% of airborne chemicals from smoke and the quantities of absorption are similar to those of smokers^29^. There is also evidence that elevated blood nicotine and cadmium levels have been observed in the population with secondhand smoke exposure^30,31^. Several studies have shown that increased serum cadmium is related to urinary tract stone formation^32–34^. Besides, nicotine from cigarette smoking could increase serum vasopressin levels, resulting in a decrease in urine output, which is a risk factor for stone formation^35–37^. Another possible mechanism is that smoking might decrease the level of calcium excretion in urine, and lower urinary calcium level is associated with increased odds of kidney stone formation^37^. Smoking can also induce oxidative stress in the kidney and lead to renal injury^38–40^, with such damage further increasing the nucleation, aggregation and retention of crystals, and promoting the formation of stones in the kidney^39,40^.

The strength of this study is being the first to demonstrate a possible influence of SHS on incident KSD. This is also a large study performed in a community-based cohort, with detailed information and regular follow-up. Despite these strengths, several limitations should be noted. Firstly, the existence of nephrolithiasis was obtained via questionnaire, without radiologic verification; however, this substitution has been validated in other studies^41,42^. Secondly, information was lacking on dietary data, but these factors are difficult to record in detail and change a lot even in individual-by-individual. Thirdly, information about type of kidney stone was lacking, but the majority of stones would be calcium oxalate stones^43^; and fourthly, there was no subgroup of children or pregnant women, because this cohort only collected adult participants, and did not mention the status of pregnancy. Finally, measurements of the toxic chemicals from SHS exposure were not performed.

In conclusion, this study suggests that SHS is a risk factor for development of kidney stones and supports the need for a prospective evaluation of this finding. Public health implications include enhancing smoke-free rules and educating the public about the adverse effects of SHS.

## Methods

### Data source and study population

This study used data from the TWB, a large-scale community-based research database comprised of cancer-free volunteers aged between 30 and 70 years enrolled through 29 recruitment centers in Taiwan since 2008. The detailed methods and profile concerning the development of TWB has been described previously^44–46^. A total of 27,209 participants with adequate baseline and long-term follow-up data were enrolled in the present study. Initially, participants with information on KSD were screened, then those with known underlying KSD (N = 1825) were excluded. Those with missing smoking data (N = 15), body mass index (N = 16), alcohol status (N = 9), physical activity status (N = 6), or serum creatinine (N = 18), and participants who were active or ex-smokers (N = 5890) were also excluded. The final analysis included 19,430 participants (Supplementary Figure S1). Participants underwent serial physical examination, biospecimen collection, and questionnaire surveys every 2 to 4 years from 2008 to 2019. The presence of a history of KSD was surveyed by a questionnaire. Written informed consent was obtained in all cases and all investigations were conducted according to the Declaration of Helsinki. This study was approved by the Institutional Review Board of Kaohsiung Medical University Hospital (KMUHIRB-E(I)-20190398).

### Smoking and SHS assessments

Firstly, participants were classified as “never-smokers”, “ex-smokers”, and “active smokers” by self-reported questionnaires. Among never-smokers, they were asked the following question: “Have you been exposed to SHS?”. Never-smokers who were exposed to SHS were assigned as the exposure group, others were allocated as the no exposure group. Participants in the exposure group were further asked “How many hours per week have you been exposed to SHS?”. According to the exposure time, we divided participants into “no exposure”, “≦ 1.2 h per week”, and “> 1.2 h per week.” The cut point of 1.2 h per week was based on the median exposure time in our cohort.

### Study outcome

In the present study, the primary end point was the development of self-reported KSD. As stated above, participants with a history of KSD were excluded. All subjects in this study had no history of kidney stone formation at baseline. During the follow-up, subjects were asked, “Have you had a kidney stone?” The development of kidney stone was defined as the subject responding “Yes” to this question. The association between SHS exposure and incident KSD was evaluated.

### Statistical analyses

Participants in the present study were stratified into no exposure and exposure groups. Clinical characteristics were presented as percentages for categorical variables, and mean ± standard deviation for continuous variables, while statistical significance of differences among categorical variables was assessed using Pearson χ^2^ test and among continuous variables were assessed using independent t-test. Logistic regression analyses were used to analyze the association of SHS exposure with incident KSD with ORs and 95% CIs before and after adjusting for confounders including age, sex, alcohol status, marital status, physical activity, BMI, SBP, DBP, history of hypertension, history of diabetes, history of dyslipidemia, hemoglobin, Hemoglobin A1c, serum fasting glucose, total cholesterol, triglyceride, low-density lipoprotein cholesterol, high-density lipoprotein cholesterol, serum albumin, serum uric acid, and estimated glomerular filtration rate. All analyses were carried out using SPSS 20.0 (IBM Corp, Armonk, NY, USA) and R version 3.6.2 (R Foundation for Statistical Computing, Wien, Austria) and a *p* value of < 0.05 was considered statistically significant.

## Supplementary Information

Supplementary Information.
