# Supplementary material for: Secondhand smoke increases the risk of developing kidney stone disease
Source: Sci Rep. 2021 Sep 6;11:17694. doi: 10.1038/s41598-021-97254-y (PMC8421344; doi:10.1038/s41598-021-97254-y)
Supplement: Supplementary file 1 — Supplementary Information. [file 41598_2021_97254_MOESM1_ESM.pdf]

Supplementary Information

Secondhand smoke increases the risk of developing kidney stone disease

Chien-Heng Chen[1], Jia-In Lee[2], Jhen-Hao Jhan[3,4,5], Yung-Chin Lee[3,4,5], Jiun-Hung Geng[3,4,5,*], Szu-Chia Chen[6,7,8,9], Chih-Hsing Hung[8,9,10,11], Chao-Hung Kuo[6,8,12]

[1]Department of Pathology, Kaohsiung Municipal Siaogang Hospital, Kaohsiung, Taiwan

[2]Department of Psychiatry, Kaohsiung Medical University Hospital, Kaohsiung Medical University, Kaohsiung, Taiwan

[3]Department of Urology, Kaohsiung Municipal Siaogang Hospital, Kaohsiung, Taiwan

[4]Department of Urology, Kaohsiung Medical University Hospital, Kaohsiung, Taiwan

[5]Kaohsiung Medical University, Kaohsiung, Taiwan

[6]Department of Internal Medicine, Kaohsiung Municipal Siaogang Hospital, Kaohsiung Medical University, Kaohsiung, Taiwan

[7]Division of Nephrology, Department of Internal Medicine, Kaohsiung Medical University Hospital, Kaohsiung Medical University, Kaohsiung, Taiwan

[8]Faculty of Medicine, College of Medicine, Kaohsiung Medical University, Kaohsiung, Taiwan

[9]Research Center for Environmental Medicine, Kaohsiung Medical University, Kaohsiung, Taiwan

[10]Department of Pediatrics, Kaohsiung Medical University Hospital, Kaohsiung Medical University, Kaohsiung, Taiwan

[11] Department of Pediatrics, Kaohsiung Municipal Siaogang Hospital, Kaohsiung Medical University, Kaohsiung, Taiwan, [12]Division of Gastroenterology, Department of Internal Medicine, Kaohsiung Medical University Hospital, Kaohsiung Medical University, Kaohsiung, Taiwan

* Correspondence author.

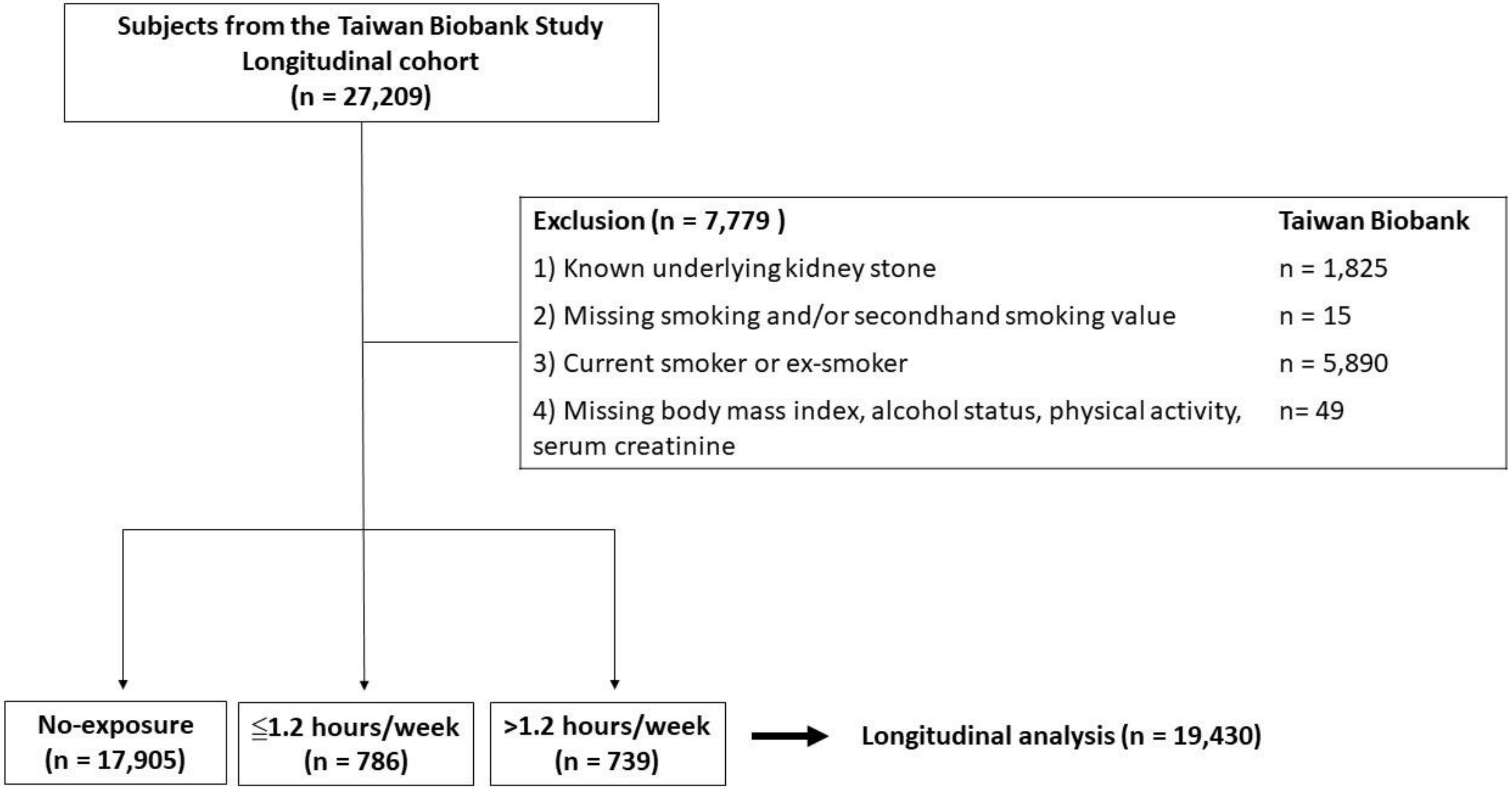

Supplementary Figure S1. Study participants were classified by secondhand smoke exposure frequency

Supplementary Table S1. Parameters associated with incident kidney stone disease in multivariate binary logistic analysis in a subgroup of participants with adequate information of daily water intake (n = 8,850)

Parameters	Odds ratio (95% CI)	*p*
Age (per 1 year)	0.997 (0.981 to 1.014)	0.767
Women (*vs.* men)	0.683 (0.448 to 1.042)	0.077
Body mass index (per 1 kg/m^2)	0.982 (0.935 to 1.030)	0.450
Married, yes (*vs.* no)	1.983 (1.022 to 3.849)	0.043
Systolic blood pressure (per 1 mmHg)	0.989 (0.975 to 1.002)	0.098
Diastolic blood pressure (per 1 mmHg)	1.034 (1.013 to 1.055)	0.001
Hypertension, yes (*vs.* no)	1.321 (0.847 to 2.061)	0.220
Dyslipidemia, yes (*vs.* no)	1.078 (0.612 to 1.898)	0.796
Hemoglobin (per 1 g/dl)	1.142 (0.999 to 1.304)	0.052
Albumin (per 1 g/dl)	0.850 (0.457 to 1.580)	0.607
Fasting glucose (per 1 g/dl)	0.992 (0.979 to 1.005)	0.234
Hemoglobin A1c (per 1 %)	1.130 (0.829 to 1.540)	0.439
Triglyceride (per 1 mg/dl)	1.001 (1.000 to 1.002)	0.093
HDL cholesterol (per 1 mg/dl)	1.001 (0.988 to 1.013)	0.928
Uric acid (per 1 mg/dl)	1.074 (0.949 to 1.215)	0.259
Daily water intake (per 1 ml)	1.000 (1.000 to 1.000)	0.590
Secondhand Smoke Exposure, yes (*vs.* no)	2.049 (1.404 to 2.990)	<0.001

BP = Blood pressure; eGFR = Estimated glomerular filtration rate; HDL = High-density lipoproteins, LDL = Low-density lipoproteins, CI = Confidence interval.

Multivariable model: adjustment for age, sex, marital status , body mass index, systolic blood pressure, diastolic blood pressure, history of hypertension, history of dyslipidemia, hemoglobin, Hemoglobin A1c, serum fasting glucose, triglyceride, high-density lipoprotein cholesterol, serum albumin, serum uric acid, and daily water intake.

Supplementary Table S2. Relative risk for incident kidney stone disease according to frequency of secondhand smoke in a subgroup of participants with adequate information of daily water intake (n = 8,850)

**Secondhand smoke frequency**	No. of Cases (%)	Number at Risk	Adjusted odds ratio (95% CI)	*P* value
No exposure	166 (2.1)	8,024	1.00 (Reference)	-
≦1.2 hours per week	16 (3.7)	434	1.69 (0.99 to 2.89)	0.055
> 1.2 hour per /week	20 (5.0)	400	2.46 (1.51 to 3.99)	<0.001

CI = Confidence interval.

Multivariable model: adjustment for age, sex, marital status , body mass index, systolic blood pressure, diastolic blood pressure, history of hypertension, history of dyslipidemia, hemoglobin, Hemoglobin A1c, serum fasting glucose, triglyceride, low-density lipoprotein cholesterol, high-density lipoprotein cholesterol, serum albumin, serum uric acid, and daily water intake.
